# CRMP5 Regulates Generation and Survival of Newborn Neurons in Olfactory and Hippocampal Neurogenic Areas of the Adult Mouse Brain

**DOI:** 10.1371/journal.pone.0023721

**Published:** 2011-10-04

**Authors:** Alexandra Veyrac, Sophie Reibel, Joëlle Sacquet, Mireille Mutin, Jean-Philippe Camdessanche, Pappachan Kolattukudy, Jérôme Honnorat, François Jourdan

**Affiliations:** 1 Team Neuroplasticity and Neuropathology of Olfactory Perception, Lyon Neuroscience Research Center INSERM U 1028/CNRS UMR 5292, Université de Lyon - Université Claude Bernard Lyon 1, Lyon, France; 2 Team Neuro-Oncology and Neuro-Inflammation, Lyon Neuroscience Research Center INSERM U 1028/CNRS UMR 5292, Université de Lyon - Université Claude Bernard Lyon 1, Faculté Laennec, Lyon, France; 3 Burnett School of Biomedical Sciences, College of Medicine, University of Central Florida, Orlando, Florida, United States of America; Universidade Federal do Rio de Janeiro, Brazil

## Abstract

The Collapsin Response Mediator Proteins (CRMPs) are highly expressed in the developing brain, and in adult brain areas that retain neurogenesis, ie: the olfactory bulb (OB) and the dentate gyrus (DG). During brain development, CRMPs are essentially involved in signaling of axon guidance and neurite outgrowth, but their functions in the adult brain remain largely unknown. CRMP5 has been initially identified as the target of auto-antibodies involved in paraneoplasic neurological diseases and further implicated in a neurite outgrowth inhibition mediated by tubulin binding. Interestingly, CRMP5 is also highly expressed in adult brain neurogenic areas where its functions have not yet been elucidated. Here we observed in both neurogenic areas of the adult mouse brain that CRMP5 was present in proliferating and post-mitotic neuroblasts, while they migrate and differentiate into mature neurons. In CRMP5^−/−^ mice, the lack of CRMP5 resulted in a significant increase of proliferation and neurogenesis, but also in an excess of apoptotic death of granule cells in the OB and DG. These findings provide the first evidence that CRMP5 is involved in the generation and survival of newly generated neurons in areas of the adult brain with a high level of activity-dependent neuronal plasticity.

## Introduction

Collapsin response mediator protein 5 (CRMP5) is a member of the collapsing response mediator protein family which is composed of five cytosolic proteins widely expressed in the brain during development, but also in some restricted areas of the adult brain [1]. These proteins have been implicated in developmental events like neuritic extension and axonal pathfinding and in some aspects of neurodegenerative processes or neuronal repair [2]–[6]. CRMP5 has been the last member of the family to be identified in the mouse [7] and rat [8], but also in man where this protein is the main target of auto-antibodies developed by patients with paraneoplasic neurological diseases [9], [10]. Like the other members of the family, CRMP5 is present in the embryonic brain [2] where its expression is consistent with a function in the permissiveness of neurite outgrowth [11], and in the regulation of filopodial dynamics and growth cone development [6], [12]. In the post-natal brain, CRMP5 is co-expressed with CRMP2 in adult oligodendrocytes where both proteins are involved in the semaphorin-3A signaling pathway [9]. CRMP5 is also present in neurons of the brain areas that retain neurogenesis, ie: the olfactory bulb (OB) [13] and the dentate gyrus (DG) of the hippocampus [14]. The subventricular zone (SVZ) of the lateral ventricles and the subgranular zone (SGZ) of the DG in the hippocampus are the two brain areas where adult neurogenesis has only been consistently found [15], [16]. In the SVZ, a subset of quiescent GFAP positive radial cells (type B cells) have the potential to serve as adult neural stem cells (NSCs) and generate transit amplifying cells (type C cells), which in turn give rise to doublecortin (DCX)-positive neuroblasts (type A cells). The neuroblasts migrate towards the OB through the rostral migratory stream (RMS), and they differentiate into olfactory bulb granular or periglomerular interneurons. In the SGZ of the DG, a population of GFAP/Nestin positive radial cells (type 1 cells) generates actively self-renewing non-radial progenitors cells (type 2 cells) which give rise to DCX-positive neuroblasts that differentiate into local glutamatergic granule cells. Many cell-intrinsic programs and extrinsic signals control neurogenesis by regulating the proliferation, fate determination, migration of progenitor cells and the long term survival and integration of mature newborn neurons [15]–[17].

The aim of the present study was to gain a better understanding of CRMP5 putative functions in neurogenic areas of the adult mouse brain, using mutant mice with a knock-out (KO) of the CRMP5 gene. We established that in both adult brain neurogenic areas, CRMP5 expression is restricted to mitotic and post-mitotic newly generated immature DCX-positive neurons. In mutant CRMP5^−/−^ mice, the number of proliferating cells and the rate of newly generated neurons were notably increased in both neurogenic areas, but this phenomenon was balanced by an excess of apoptotic neuronal death. Our results suggest that CRMP5 is most likely involved in intracellular pathways which determine the rate of generation and survival of newly generated neurons in the two adult brain neurogenic areas.

## Results

### CRMP5 is constitutively expressed by doublecortin-positive neuroblasts in the adult brain neurogenic areas

**Figure 1:**
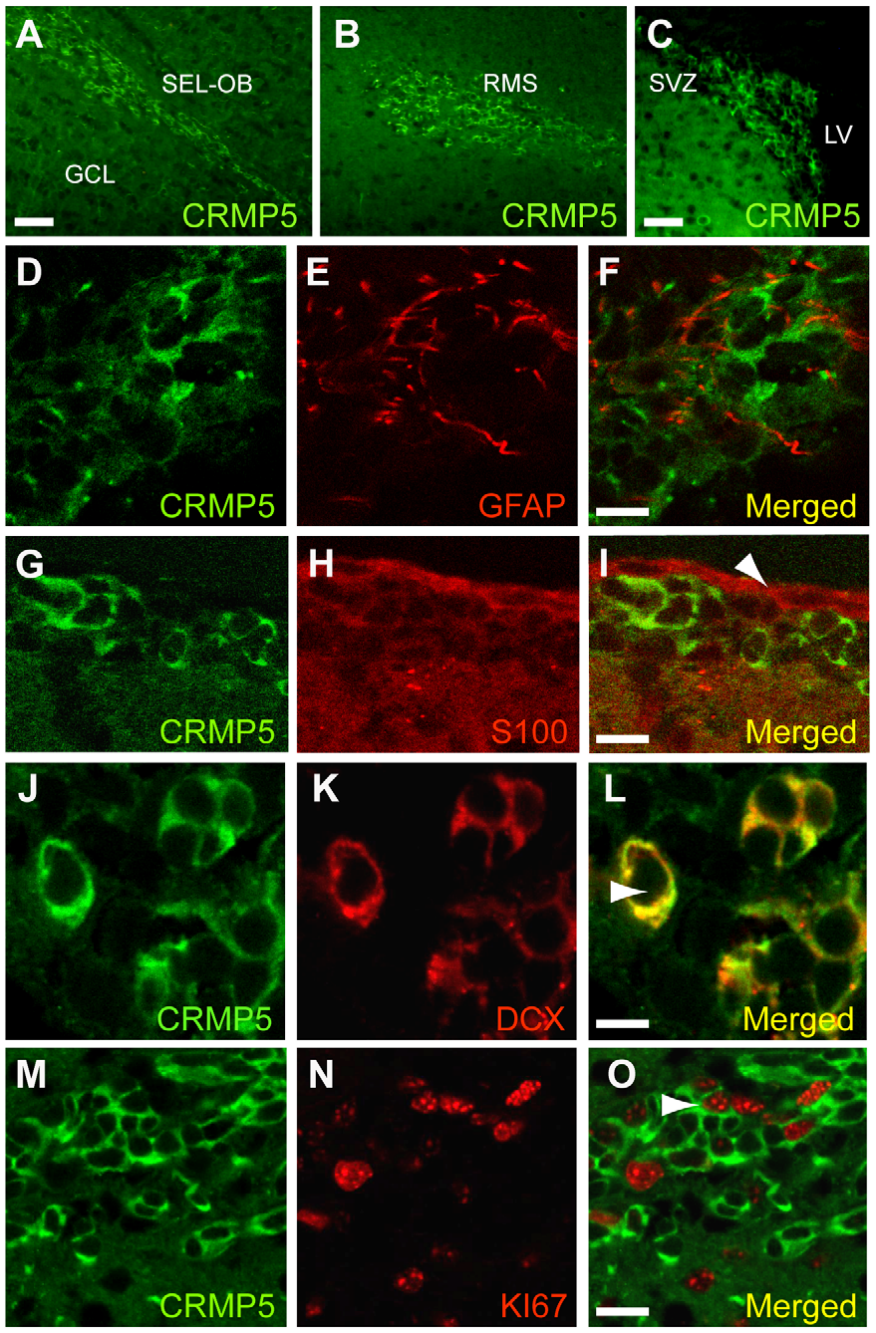
Immunofluorescence labeling of CRMP5 expression in neuroblasts of the mouse olfactory neurogenic area. (**A–C**) Confocal microscopy of immuno-labelled sections showing numerous CRMP5-positive cells at three levels of the olfactory neurogenic area, ie: the subependymal layer of the OB (SEL-OB), the rostral migratory stream (RMS) and the subventricular zone (SVZ) of the forebrain. (**D–O**) Characterization of CRMP5-positive cells in the SVZ. Double immunolabeling of CRMP5 and the glial markers GFAP (**D–F**) or S100 (**G–I**, arrow) revealed the lack of CRMP5 expression in glial cells. In contrast, all CRMP5-positive cells co-expressed doublecortin (DCX) a selective marker of immature neurons (or neuroblasts) (**J–L**, arrow). Finally, double immunolabeling of CRMP5 and KI67, a marker of cycling cells, shows that some CRMP5-positive cells are still mitotic (**M–O**, arrow), whereas the majority are no longer proliferative. Lateral ventricles (LV). Optical thickness of the confocal planes = 1 µm. Scale bars 50 µm (A–C); 15 µm (D–I); 10 µm (J–L); 20 µm (M–O).

In the adult mouse forebrain, cells of the neuronal lineage proliferate in the SVZ, migrate along the RMS and reach the OB where they differentiate into interneurons [15]. As shown by CRMP5 immunolabeling (Fig. 1), numerous densely-packed CRMP5-positive cells are present at every level of the olfactory neurogenic area, ie: the subependymal layer (SEL) of the OB (Fig. 1A), the RMS (Fig. 1B), and the SVZ (Fig. 1C). In the SVZ, double immunolabeling shows that CRMP5 was neither co-expressed with the glial markers GFAP (Fig. 1 D–F) or S100 (Fig. 1 G–I), nor with Nestin, a marker of transit amplifying (type “C”) cells (data not shown). By contrast, CRMP5 in the SVZ was systematically co-localized with the neuroblast markers doublecortin (DCX) (Fig. 1 J–L), PSA-NCAM and TUC4/CRMP4 (data not shown). Since the SVZ contains cell populations with different rates of proliferation, we performed a double immunolabeling of CRMP5 with the cycling-cell marker KI67. As shown in Figure 1 M–O, a sub-population (17.8±3.6%) of CRMP5-expressing cells is still cycling (KI67-positive). These CRMP5-positive cycling cells represented 48.3±9.6% of the entire population of KI67-positive proliferating cells in the SVZ.

**Figure 2:**
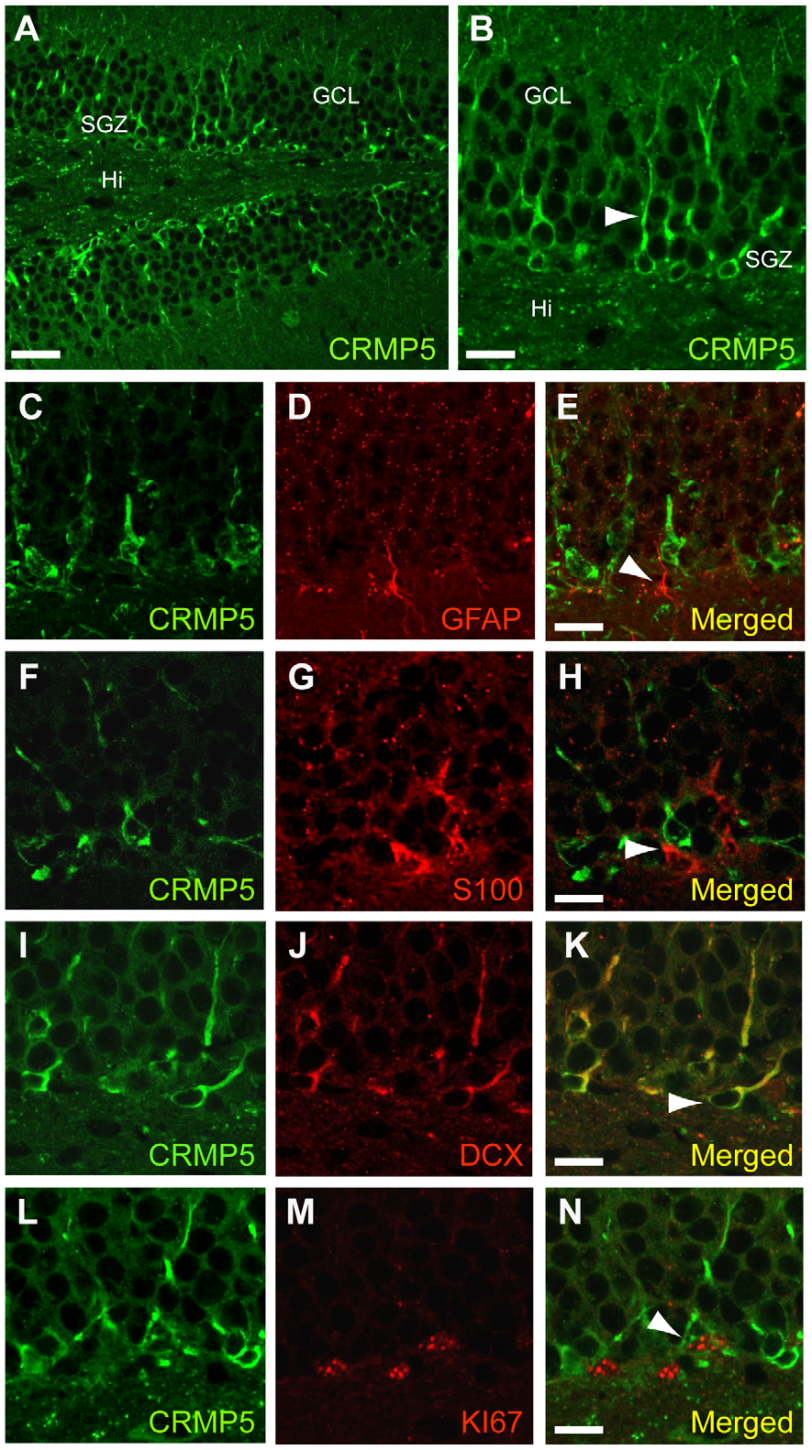
Immunofluorescence labeling of CRMP5 expression in neuroblasts of the mouse hippocampal neurogenic area. (**A,B**) Confocal microscopy of immuno-labelled sections showing CRMP5-positive cells in the subgranular zone (SGZ) of the dentate gyrus (DG) in the hippocampus. (**C–N**) Double immunolabeling in the DG of CRMP5 with the glial markers GFAP (**C–E**) and S100 (**F–H**) demonstrates that glial cells never expressed CRMP5 (2E and 2F, arrows). In contrast, all CRMP5-positive cells co-expressed doublecortin (DCX) a selective marker of immature neurons (or neuroblasts) (**I–K**, arrow). Finally, double immunolabeling of CRMP5 and KI67, a marker of cycling cells, shows that some CRMP5-positive cells are still mitotic (**L–N**, arrow), whereas others are no longer proliferative. Granular cell layer (GCL); Hilus (Hi). Optical thickness of the confocal planes = 1 µm. Scale bars 50 µm (A,B); 20 µm (C–E); 15 µm (F–N).

In the adult hippocampus, CRMP5 is highly expressed in the SGZ of the DG (Fig. 2 A, B). As already observed in the SVZ, double immunolabeling of CRMP5 with markers of glial cells (GFAP/S100; Fig. 2 C–E and 2 F–H) or transit amplifying cells (Nestin; data not shown) failed to reveal any co-expression. By contrast, all CRMP5-positive cells systematically co-expressed the neuroblast specific markers DCX (Fig. 2 I–K), PSA-NCAM and TUC4/CRMP4 (data not shown). Furthermore, double labeling of CRMP5 with KI67, a marker of cycling cells, (Fig. 2 L–N) revealed that 30.3±5.4% of CRMP5-positive cells co-expressed KI67 and were still proliferating in the DG, whereas 43.9±9.4% of KI67-positive cells were CRMP5-positive.

Altogether, our findings indicate that in both adult forebrain neurogenic areas, CRMP5 expression is restricted to mitotic and post-mitotic neuroblasts, the most numerous CRMP5-positive neuroblasts being post-mitotic cells probably engaged in the migration and differentiation processes.

### Suppression of CRMP5 expression increases cell proliferation in the adult brain neurogenic areas

**Figure 3:**
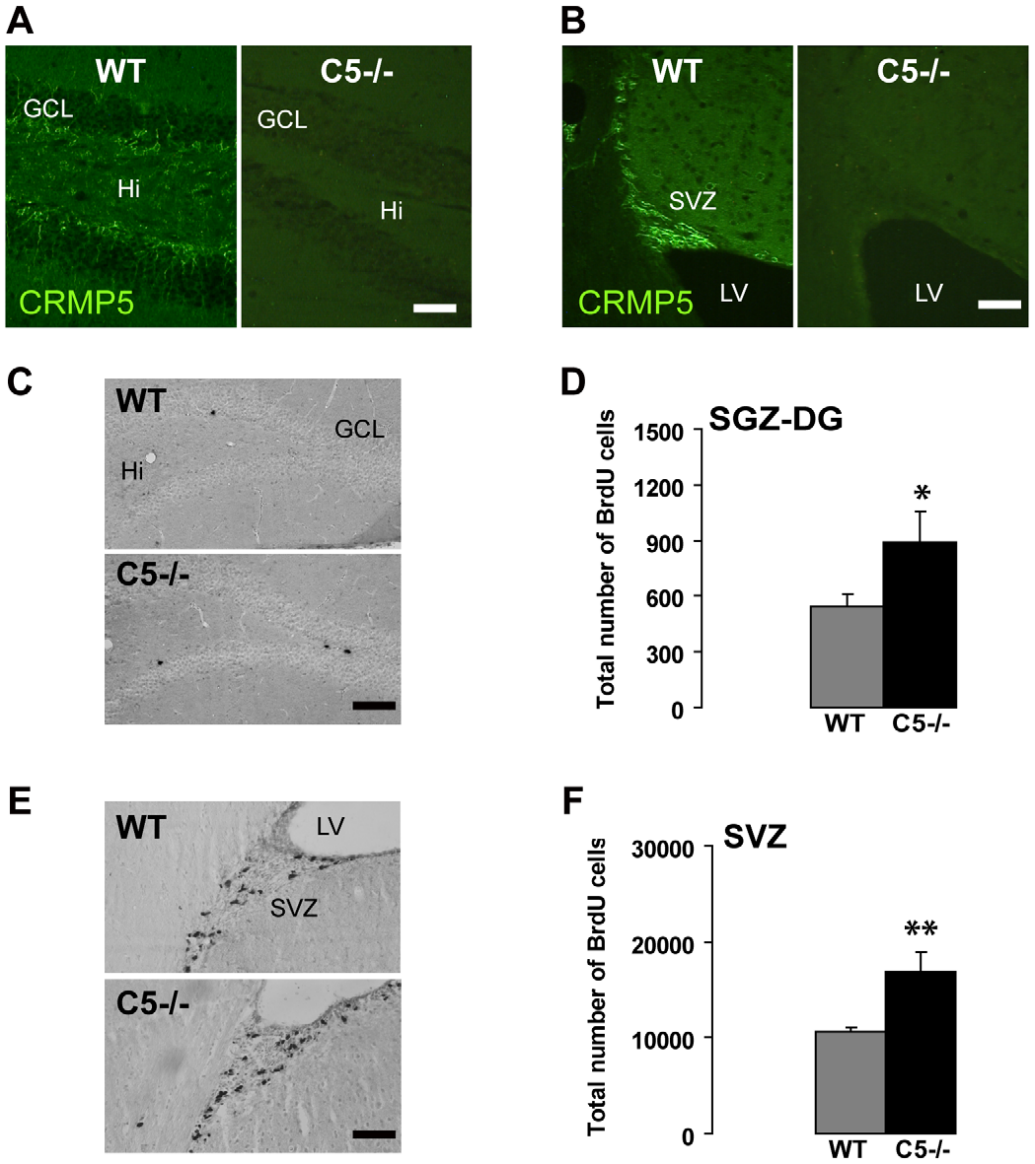
Suppression of CRMP5 expression in CRMP5^−/−^ mice increases cell proliferation in the adult brain neurogenic areas. (**A,B**) Immunofluorescence detection of CRMP5 in the dentate gyrus (3A) and SVZ (3B) confirms the lack of CRMP5 expression in CRMP5^−/−^ mice in comparison to wild type mice (WT). (**C**) Immunocytochemical labeling of BrdU-positive cells in the SGZ of the dentate gyrus (DG) of WT and CRMP5^−/−^ mice, 2 hours after BrdU injections. Hilus (Hi); Granular cell layer (GCL). Scale bar 100 µm. (**D**) Increased total number of BrdU positive cells in the DG of CRMP5^−/−^ mice. Values are expressed as mean + SEM. Student t-test. * p<0.05; ** p<0.01. (**E**) Immunocytochemical labeling of BrdU-positive cells in the subventricular zone (SVZ) of WT and CRMP5^−/−^ mice, 2 hours after BrdU injections. Lateral ventricles (LV). Scale bar 100 µm. (**F**) Increased total number of BrdU positive cells in the SVZ of CRMP5^−/−^ mice. Values are expressed as mean + SEM. Student t-test. * p<0.05; ** p<0.01.

Since CRMP5 is highly expressed in neuroblasts of both neurogenic areas, including a sub-population of proliferating cells, we have assessed cell proliferation in the adult brain neurogenic areas of mice with a KO of the CRMP5 gene (CRMP5^−/−^ mice) (Fig. 3 A, B). Following a short-pulsed BrdU incorporation assay (2 hours), the number of proliferating BrdU-positive cells was significantly increased in both neurogenic areas in CRMP5^−/−^ mice when compared with wild type (WT) mice (Fig. 3 C–F) (SGZ: Fig. 3D t-test p = 0.045 / SVZ Fig. 3 F; t-test p = 0.008). These data show that CRMP5 takes part to the negative regulation of the rate of cell proliferation in both adult brain neurogenic areas.

### Suppression of CRMP 5 expression increases the number of newly generated neurons in the adult olfactory bulb and dentate gyrus

**Figure 4:**
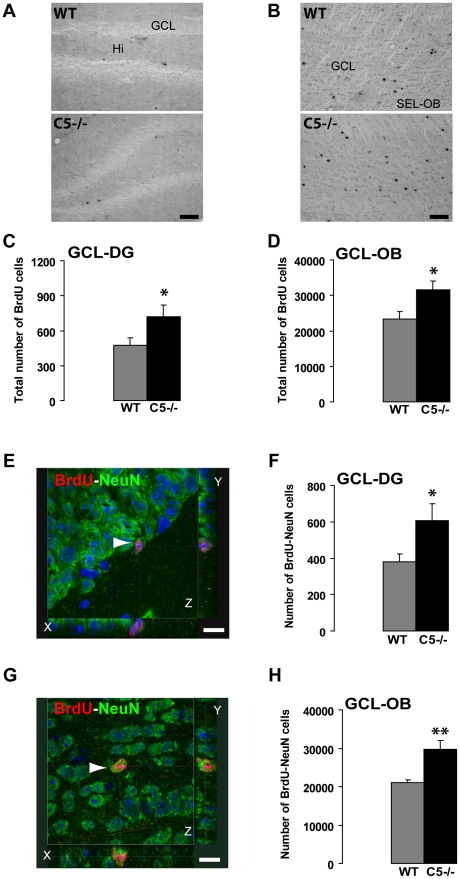
Suppression of CRMP5 expression increases the rate of newly generated neurons in the adult olfactory bulb and dentate gyrus of CRMP5^−/−^ mice. (**A,B**) Immunocytochemical labeling of BrdU-positive cells in the DG (A) and in the OB (B) of WT and CRMP5^−/−^ mice, 21 days after BrdU injections. Hilus (Hi); Granular cell layer (GCL); Subependymal layer (SEL). Scale bar 100 µm. (**C,D**) Increased total number of BrdU positive cells in the DG (**C**) and in the OB (**D**) of CRMP5^−/−^ mice. Values are expressed as mean + SEM. Student t-test. * p<0.05; ** p<0.01. (**E**). Immunocytochemical labeling of BrdU-NeuN double-labeled cell in the DG of WT mouse (arrow, step size of confocal Z planes = 1 µm). Scale bar 15 µm. (**F**). Total number of BrdU-NeuN positive cells is increased in the DG of CRMP5^−/−^ mice. Values are expressed as mean + SEM. Student t-test. * p<0.05; ** p<0.01. (**G**). Immunocytochemical labeling of BrdU-NeuN double-labeled cell in the GCL of the OB of WT mouse (arrow, step size of confocal Z planes = 1 µm). Scale bar 15 µm. (**H**). Total number of BrdU-NeuN positive cells is increased in the OB of CRMP5^−/−^ mice. Values are expressed as mean + SEM. Student t-test. * p<0.05; ** p<0.01.

The CRMP5-positive neuroblasts generated in the adult neurogenic areas give rise to newborn neurons which migrate, survive and integrate the neuronal networks of the OB and DG. So, we have investigated the rate of neurogenesis in WT and CRMP5^−/−^ mice by analyzing the number of newly generated BrdU-labeled cells in the OB and the DG, 21 days after BrdU administration. We found that the number of BrdU-labeled newborn cells was higher in the CRMP5^−/−^ mice compared to WT in both the DG (+51%; t-test p = 0.015; Fig. 4 A,C) and the OB (+35%; t-test p = 0.038; Fig. 4 B,D). In order to test the contingent effect of the lack of CRMP5 on the fate of these newborn cells, we assessed the number of BrdU-positive cells co-localizing the neuronal marker NeuN in the DG (Fig. 4 E,F) and the OB (Fig. 4 G,H). In both cases, the number of newborn neurons increased significantly in CRMP5^−/−^ mice (DG: +60%, t-test p = 0.027, Fig. 4 E,F; OB: +41%, t-test p = 0.005, Fig. 4 G,H), indicating that neurogenesis was stimulated in both neurogenic areas. However, the proportion of 21 days-old BrdU-positive cells with a neuronal phenotype (co-expressing the neuronal marker NeuN) was not significantly modified in CRMP5^−/−^ mice (OB: 90.8±3.6% in WT versus 94.7±2.8% in KO mice, p = 0.211; GD: 83.5±7.7% in WT versus 80.9±6.2% in KO mice, p = 0.40; data not shown). As a consequence, these results show that the lack of CRMP5 stimulated neurogenesis without affecting significantly the ratio of neuronal versus non-neuronal newborn cells in the target areas.

### Suppression of CRMP5 expression increases neuronal cell death in the olfactory bulb and dentate gyrus

**Figure 5:**
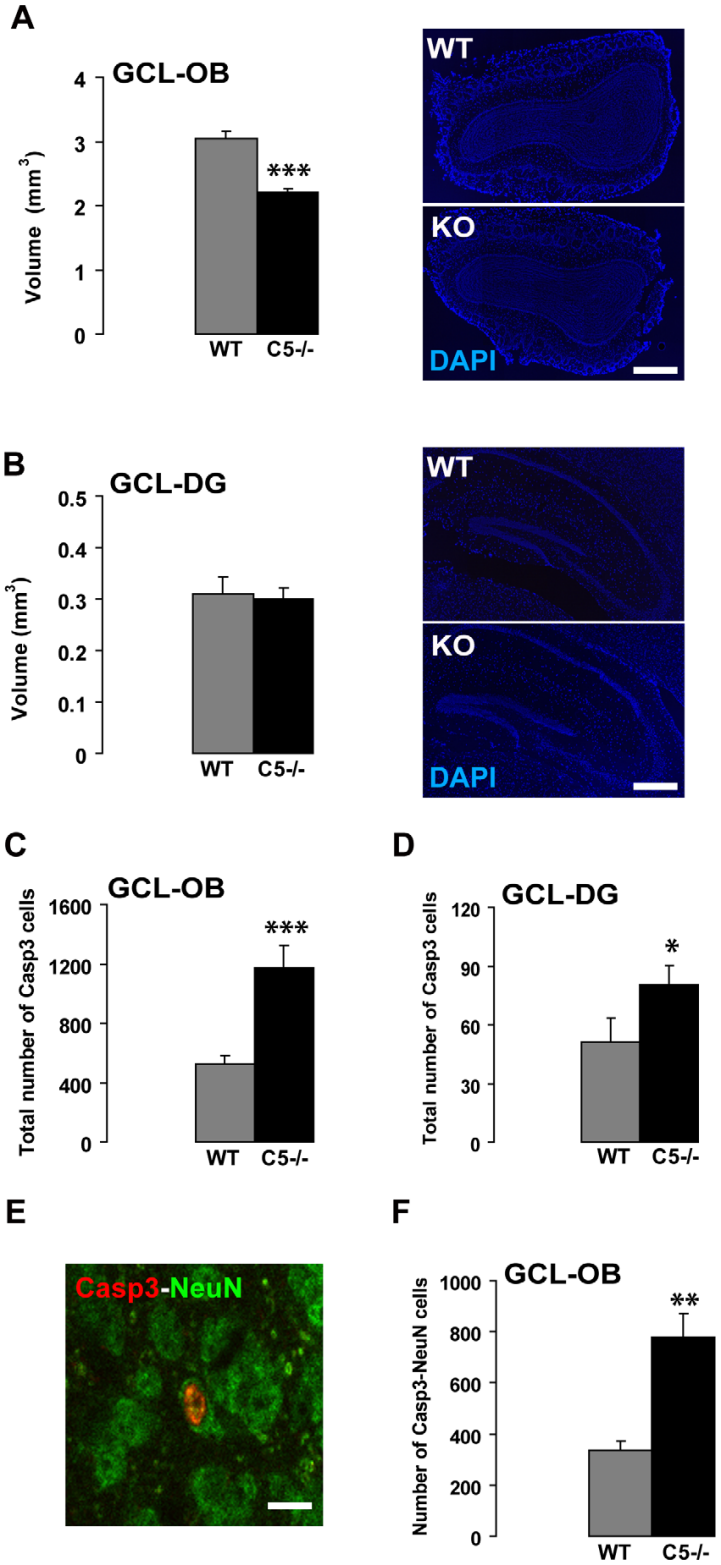
Suppression of CRMP5 expression increases neuronal cell death in the olfactory bulb and dentate gyrus of CRMP5^−/−^ mice. (**A,B**) Stereological measurements of the volume of granular cell layers in the OB (A) and the DG (B) of wild type (WT) and CRMP5^−/−^ mice. In the OB (A), the lack of CRMP5 in CRMP5^−/−^ mice induced a significant reduction of the granule cell layer thickness (*** p<0.005) when compared to the wild type. DAPI-stained coronal sections of the OB failed to reveal other major morphological alterations in KO mice (Scale bar 400 µm). In the DG (B), the thickness of the granule cell layer was similar in the CRMP5^−/−^ and wild type mice. DAPI-stained coronal sections of the dorsal hippocampus failed to reveal any major morphological alterations in CRMP5^−/−^ mice (Scale bar 400 µm). All values are expressed as mean + SEM. Student t-test: *** p<0.005. (**C,D**). Increased number of caspase-3 positive (apoptotic) cells in the OB (**C**) and in the DG (**D**) of CRMP5^−/−^ mice when compared to the wild type (WT). Values are expressed as mean + SEM. Student t-test. * p<0.05; *** p<0.005. (**E**). Confocal microscopy of a caspase-3/NeuN immunolabeled cell (apoptotic neuron) in the DG of WT mouse (arrow, step size of confocal Z planes = 1 µm). Scale bar 15 µm. (**F**). Increased total number of caspase-3/NeuN immunoreactive cells in the OB of CRMP5^−/−^ mice when compared to wild type (WT) mice. Values are expressed as mean + SEM. Student t-test. ** p<0.01.

Following the observation of an increased rate of neurogenesis in CRMP5^−/−^ mice, and given the regulated balance between neuronal proliferation and death in the OB [18] and the DG [19], we later assessed the putative effects of the lack of CRMP5 on cell apoptosis in both neurogenic areas of the adult brain. The rate of cell death has been estimated using the cleaved form of caspase-3 as a reliable marker of apoptosis [20], [21]. In addition, the volumes of the OB and DG granule cell layers have been measured and compared between WT and CRMP5^−/−^ mice. In CRMP5^−/−^ mice, we observed a significant volume decrease (about 30%) of the OB granule cell layer (Fig. 5A; t-test p = 0.00014), whereas the DG granule cell layer remained unaffected (Fig. 5B; t-test p =  0.420). In accordance with this volume decrease, the number of apoptotic (caspase-3 positive) cells in the OB granule cell layer was significantly higher in CRMP5^−/−^ mice compared to the WT (Fig. 5C; t-test p = 0.0018). In the DG granule cell layer of CRMP5^−/−^ mice, a less intense but significant increase of apoptotic cell number was also observed (Fig. 5D; t-test p =  0.049), in spite of the unchanged global volume of the layer (Fig. 5B).

In order to assess the rate of neuronal death, we estimated by double immunolabeling the number of cells co-expressing caspase-3 and the neuronal marker NeuN. In the OB of CRMP5^−/−^ mice, we found a strong increase of the absolute number of dying neurons (caspase-3/NeuN-positive) (Fig. 5E, F; t-test p = 0.007) without modification of the ratio of neuronal vs non-neuronal phenotypes in apoptotic cells (percentage of caspase-3/NeuN positive cells in WT: 69.1±0.9% versus 67.4±4.8% in KO mice, p = 0.21). As a consequence of neuronal degeneration in the OB granule cell layer of CRMP5^−/−^ mice, we found a significant decrease of the area of NeuN-immunoreactive profiles (relative to the total area) in this layer (WT: 0.32±0.007% versus KO: 0.30±0.01%; t-test p = 0.049; data not shown).

In the DG we could not observe any dying cells (caspase3-positive) co-expressing NeuN, suggesting that neurons died before becoming mature in this area.

## Discussion

### CRMP5 expression in neurogenic areas of the adult brain

In both neurogenic areas of the adult brain, the expression of CRMP5 is restricted to a minor fraction of neuroblasts that continue to divide (about 18% in the SVZ and 30% in the SGZ) and to all the post-mitotic neuroblasts while they migrate and reach their targets (OB and DG respectively). This observation is reminiscent of our previous finding that CRMP5 was the most abundant CRMP expressed by immature neurons in the adult mouse OB and DG [9], [13], [14]. However, contrary to CRMP1 or CRMP2, CRMP5 expression is down-regulated when newly generated neurons reach maturity [13], [14]. Furthermore, CRMP5 is temporally and spatially co-expressed with DCX, PSA-NCAM and TUC/CRMP4 which are considered as reliable markers of neuroblasts and immature neurons in adult neurogenic areas [15], [17], [22]. These statements lead us to the proposal that CRMP5 could be further used as a reliable marker of immature cells of the neuronal lineages in the adult brain, at least in the olfactory and hippocampal neurogenic areas.

The restriction of CRMP5 expression to immature newborn neurons in the adult neurogenic areas supports the previous assumption that this protein was involved in neuronal differentiation, at both developmental and adult stages [12], [13]. However, about 18% of CRMP5-expressing cells in the SVZ and 30% in the SGZ were still cycling and most likely belong to a transit amplifying neuroblast population. This original finding is in favor of a role of CRMP5 in the transition phase between proliferative and non-proliferative stages, suggesting that CRMP5 might play an active role in adult neurogenesis by taking part to the repression of proliferation and/or to the induction of the first stages of neuronal differentiation. The high expression level of CRMP5 in aggressive neuroendocrine tumors [23] highlights the likely involvement of this protein in the balance between proliferation and differentiation in different cell types and in both physiological and pathological conditions.

### CRMP5 knock-out and adult neurogenesis: role of CRMP5 in proliferation, differentiation and survival of neurons

Adult neurogenesis is a dynamic process regulated by a finely tuned balance between sequential events like stem cell and progenitors proliferation, migration, differentiation, and cell death or survival of neuroblasts and immature neurons (review in [15]). In CRMP5^−/−^ mice, the proliferation of progenitors and the number of migrating neuroblasts increased significantly (and in similar proportions) in both neurogenic areas. Complementary to the observation that CRMP5 expression starts at the latest stages of proliferation in WT mice, this finding strongly suggests that CRMP5 could participate to intracellular signaling pathways that regulate negatively the proliferation of transient amplifying cells and/or young neuroblasts. Since the increased number of newborn neurons (NeuN-positive) in the OB of CRMP5^−/−^ mice was similar to the excess of neuroblasts in their SVZ, the lack of CRMP5 has not interfered with the migration of neuroblasts towards the olfactory bulb. Thus, we may assume that CRMP5 is most likely not involved in the regulation of migration of neuroblasts and immature neurons in the adult brain. The fate of neural progenitors (neuronal versus non-neuronal lineages) seems no more dependent of CRMP5 since the ratio of these phenotypes was not altered in newly generated cells of CRMP5^−/−^ mice.

It is worth noting that the overflow of newborn neurons in neurogenic areas of adult CRMP5^−/−^ mice was followed by a strong increase of apoptotic neuronal death in target areas, and even a significant volume decrease of the granule cell layer in the OB. In the DG, the increased rate of cell death did not result in a decrease of the granule cell layer volume, probably due to the lower rate of neurogenesis in this cortex and its reduced structural impact [24].

Our observation of a strong increase of neuronal apoptosis in CRMP5^−/−^ mice argues for the involvement of CRMP5 in the regulation of their survival. In the OB of WT mice, we have previously shown that CRMP5 is mainly restricted to cell bodies of neuroblasts lying in the subventricular zone and to the apical dendrites of bulbar immature interneurons at the earliest stages of their radial migration and differentiation [13]. Thus, we might assume that CRMP5 could be essential for a differentiation process determining the ability of immature interneurons to integrate the local neuronal networks for becoming functional and survive. This assumption is supported by the recent demonstration of aberrant morphology and reduced diameter of Purkinje cell dendrites from post-natal day 21 in CRMP5^−/−^ mice [25]. Thus, the post-natal maturation of Purkinje cells, as the maturation of newly generated interneurons in the adult OB, seems most likely submitted to CRMP5-dependent mechanisms occurring in their dendrites.

The rise of neuronal apoptosis in CRMP5^−/−^ mice must be interpreted relative to the increase of proliferation and to the resulting excess of incoming neuroblasts in the OB and DG. In the cerebellum of juvenile CRMP1^−/−^ mice, the lack of CRMP1 induced a decrease of granule cell proliferation and a strong reduction of the apoptosis of newborn neurons [26]. Although CRMP1 and CRMP5 deficiencies resulted in opposite effects on neurogenesis (via proliferation and death) in the juvenile cerebellum and adult neurogenic areas respectively, proliferation and apoptosis of newborn neurons are similarly co-regulated in both cases, in order to maintain a steady population of newly generated neurons. The expression profiles of CRMP1 and CRMP5 have been shown to overlap accurately during brain development, although CRMP5 cannot interact directly with CRMP1 [7]. In the adult OB, CRMP1 has been co-localized with CRMP5 in the neuroblasts of the olfactory subependymal layer, but CRMP5 concentrates in the apical dendrites of immature newborn granule cells while CRMP1 remains mainly in their pericarya [13]. Thus, CRMP5 and CRMP1 might contribute synergistically to the regulation of the balance between functional integration and death of newborn neurons in the adult neurogenic areas, via pathways excluding direct molecular interaction between these two proteins.

The contribution of CRMP5 to the maturation of newborn neurons in neurogenic areas might be crucial for local information processing in the DG and OB since CRMP5^−/−^ mice display impairments of long-term depression in Purkinje cells and behavioral motor disorders [25]. Furthermore, CRMP1^−/−^ mice exhibit impaired long-term potentiation in the hippocampus and spatial learning deficits [27]. These data argue for a major role of CRMPs, including CRMP5, in dendritic plasticity, with important implications for the maturation and functionality of networks involving newborn neurons in neurogenic areas of the adult brain.

### Mechanisms of CRMP5 involvement in adult neurogenesis

The present study provides the first evidence that CRMP5 is a likely key intracellular signal of neurogenesis regulation, not only during brain development, but also in neurogenic areas of the adult brain. CRMP5, the most recently identified member of the CRMPs family [7]–[9], is strongly expressed in the developing brain, but is restricted to neurogenic areas in the adult brain [9], [13]. The spatial and temporal patterns of CRMP5 expression led authors to assume its functional involvement in neuronal migration/differentiation [9], and more specifically in axonal growth during development [11], [12]. The present study emphasizes the role of CRMP5 in the balanced regulation of neuroblast proliferation and survival of immature newly generated neurons, in the DG and OB of the adult brain. Whether these functions mimic those assumed by CRMP5 in the developing brain remains to be elucidated, but we can assume that axonal growth is not a major functional target of CRMP5 in the olfactory neurogenic area since the main population of CRMP5-positive immature newborn neurons differentiate into granule cell interneurons which belong to the family of amacrine interneurons lacking a typical axon. By contrast, CRMP5 concentrates in the apical dendrite of immature granule cells and probably contributes to the maturation and synaptic connectivity of these dendrites which play a major role in the local processing of sensory information [18].

How can CRMP5 contribute to the proliferation, plasticity and survival of newly generated neurons in the DG and OB? First, we should notice that CRMP5 is co-expressed with the other members of the CRMP family, CRMP1, CRMP2 [13] and CRMP4 [2], in neuroblasts and immature neurons of adult brain neurogenic areas. As discussed above, CRMP1 and CRMP5 have opposite effects on proliferation and survival of newborn neurons, and they might contribute to the balance between neuronal proliferation and death through different pathways, since they cannot interfere with each other [6]. By contrast, interactions between CRMP5 and CRMP2 are most likely to occur. CRMP2 is the best known CRMP, and several studies demonstrated its involvement in the promotion of axonal growth and neuronal polarity through direct binding to tubulin and promoting microtubule dynamics [28], [29]. CRMP5 has been often found associated with CRMP2 and it has recently been shown to display opposite capacities, like inhibition of tubulin polymerization and neurite outgrowth [6]. Moreover, CRMP5 is able to abrogate the neurite outgrowth promotion induced by CRMP2, as a dominant signal [6]. Thus, CRMP2 and CRMP5 have antagonistic effects on crucial cellular events related to neuronal differentiation and maturation, and probably take part synergistically to these processes in both neurogenic areas of the adult brain. As a dominant signal, CRMP5 likely plays a major role in the regulation of adult neurogenesis by other CRMPs, particularly CRMP2.

Indeed, many intrinsic and extrinsic factors have been identified as potential regulators of neural proliferation and/or neuronal differentiation in the adult OB or DG [15]. In addition to a CRMP5 influence on proliferation or differentiation through a direct impact on cytoskeletal proteins, or through a modulation of other CRMPs, we cannot rule out the possibility that CRMP5 might interact with other signals like neurotrophins. The brain-derived neurotrophic factor (BDNF) has long been identified as playing several crucial functions in neuron survival, dendritic development or neuronal plasticity, in both the adult neurogenic areas, SGZ [30] and SVZ [31]. A study using CRMP5^−/−^ mice has recently brought support to a possible interaction between CRMP5 and BDNF in the regulation of adult neurogenesis [25]. The stimulating effect of BDNF was impaired in cultured hippocampal neurons from CRMP5^−/−^ mice, and CRMP5 was tyrosine phosphorylated by the high affinity BDNF receptor, TrKB. However, further *in vivo* studies are now needed for the elucidation of CRMP5 involvement in the regulation of BDNF impact on neurogenesis and neuronal differentiation in the adult brain neurogenic areas.

Finally, the present study provides the first evidence that CRMP5 is a likely key intracellular signal of neurogenesis regulation, not only during brain development, but also in neurogenic areas of the adult brain. CRMP5 is a negative regulator of the proliferation of neural progenitors and transit amplifying neuroblasts, contrary to CRMP1 which stimulates proliferation. However, CRMP5 likely takes part to neuronal differentiation events which facilitate the integration of newborn neurons in the local networks, and their survival, through a direct action on cytoskeletal proteins. In addition, CRMP5 could also interfere with CRMP2 effects on neurite outgrowth or other aspects of neuronal differentiation. Further studies should contribute to elucidate other signaling pathways in which CRMP5 could participate, and to assess the functional relevance of its expression in activity-dependent plasticity that occurs in the olfactory bulb and the hippocampus.

## Materials and Methods

### Generation of CRMP5^−/−^ mice

Mice deficient in the CRMP5 gene were generated as described previously [25]. Heterozygous animals were identified by genotyping, and bred together to produce homozygous mutant mice. The CRMP5^−/−^ mice appeared normal, without any obvious gross abnormalities, and they reproduced normally. The absence of expression of CRMP5 was confirmed in these animals by immunohistochemistry, immunoblot analysis and RT-PCR analyses [25]. All animals were housed under a 12-h light/dark cycle with food and water available *ad libitum*. All efforts were made to minimize the number of animals used and their suffering during experimental procedure in accordance with NIH guidelines concerning the Care and Use of Laboratory Animals and with the approval of the Institutional Animal Care and Use Committee of the University of Central Florida.

### BrdU administration

10 adult 12-week-old male mice (5 WT and 5 CRMP5^−/−^) received two injections, 2 hours apart, of 5-bromo-2′deoxyuridine (BrdU Sigma Saint Louis MO; 50 mg/kg in physiological saline) 2 hours before killing, to assess cell proliferation. To assess newborn cell survival, another series of 10 mice (5 WT and 5 CRMP5^−/−^) received three BrdU injections every 2 hours before being returned to their respective cages for the remaining 21 days of the experiment.

### Tissue processing and sectioning

Animals were deeply anesthetized with isoflurane gas and perfused transcardially with a fixative solution of 4% paraformaldehyde in 0.1 M phosphate buffer, pH 7.4 (PB). Brains were dissected out and fixed overnight at 4°C, washed 12 h in PB and immersed 72 h at 4°C in PB containing 20% sucrose, then frozen in chilled isopentane (−55°C). Coronal sections (14 µm) were made in a cryostat (Leica) and collected on Superfrost Plus slides.

### Immunohistochemistry

#### CRMP5 double labeling

For double immunolabeling associating KI67 or Doublecortin (DCX) with CRMP5, brain sections were firstly heated for 20 min at 98°C in Target Retrieval Solution (DAKO, Trappes, France) before being pretreated 90 min with blocking solution (2% BSA, 0.3% Triton, 5% serum) and incubated overnight at 4°C with primary antibodies. The primary antibodies used in this study were the rabbit anti-CRMP5 antibody (2 µg/ml) previously manufactured in our laboratory [4], [9], [13], rabbit anti- CRMP4 (1/500; Chemicon, Millipore, Billerica, MA, USA), guinea pig anti-DCX (1/1000; Chemicon, Millipore, Billerica, MA, USA), mouse anti-PSA-NCAM (1/100; AbCys, Paris, France), mouse anti-KI67 (1/100, Novacastra, Newcastle,UK), mouse anti-GFAP (1/200, DAKO), mouse anti-S100β (1/400, Sigma) and mouse anti-Nestin (1/200, Pharmingen, San Lose, CA). Finally, the sections were incubated for 2 hours in species-specific secondary antibodies conjugated to Alexa 488, 546 or 633 (Molecular Probes, Invitrogen, Eugene, OR, USA), for immunofluorescence labeling.

#### BrdU and Caspase 3 labeling

For BrdU immunocytochemistry, brain sections were pre-incubated in Target Retrieval Solution for 20 min at 98°C. After cooling for 20 min, sections were treated with Triton 0.5% in phosphate-buffered saline (PBS) for 30 min, then for 3 min with pepsin (0.43 U/ml in 0.1 N HCl; Sigma). Further, endogenous peroxidases were blocked with a solution of 3% H2O2 in 0.1 M PBS and the sections were then incubated for 90 min in 5% normal serum (Vector Laboratories), 5% bovine serum albumin (Sigma) and 0.125% Triton X-100 to block nonspecific binding. BrdU or Caspase 3 immunolabeling were obtained by incubating the sections overnight at 4°C in a mouse anti-BrdU primary antibody (1/100; Chemicon) or 48 h at 4°C in a Rabbit Anti-Caspase 3 (Casp3) (1/500; Cell Signaling Technology). For BrdU detection, the sections were then incubated for 2 h at room temperature in a horse biotinylated anti-mouse (1/200; Vector Laboratories) whereas for caspase 3 detection they were incubated in a goat anti-rabbit (1/200; Vector Laboratories) secondary antibody. The sections were then processed with avidin-biotin-peroxidase complex (ABC Elite Kit; Vector Laboratories) for 30 min, followed by three rinses of 5 min in PBS. Finally, they were reacted in 0.05% 3,30-diaminobenzidine-tetrahydrochloride (Sigma), 0.03% NiCl2, and 0.03% H2O2 in Tris-HCl buffer (0.05 M, pH 7.6), dehydrated in graded ethanols, and coverslipped in DPX.

For BrdU and Casp3 double-labeling experiments, the sections were treated as described before except that they were incubated simultaneously with mouse anti-NeuN antibodies (1/500; Chemicon), guinea pig anti-DCX (1/1000), mouse anti-GFAP (1/200) or rat anti-BrdU (1/100; Oxford Biotechnology, Abcys) at 4°C overnight. Immunoreactivities were revealed for 2 hours using species-specific secondary antibodies conjugated to Alexa 488, 546 or 633 or biotinylated secondary antibodies followed by streptavidin Alexa 488 (Molecular probe).

### Quantification and image analysis

#### Number and density of BrdU- and Casp3-labelled cells

Immunolabeled profiles were counted in 14 µm-thick coronal sections under a Zeiss microscope coupled with a specific mapping software (Mercator Pro; Explora Nova, La Rochelle, France). The brain areas of interest were the granular, and subependymal layers of the OB (five sections per animal, inter-section intervals of 336 mm), the granular layer of the DG (five to seven sections per animal, inter-section intervals of 336 mm) and the forebrain SVZ (four sections per animal; inter-section intervals of 336 mm). The volume of each brain area was calculated according to a conventional stereological equation [32] as described previously [20]. The density (number of cells/µm^2^) and the total number of labeled cells were inferred from these data as described previously. All the data were averaged across animals within each experimental group and statistical differences were assessed using a Student t-test.

#### NeuN labeling analysis

Images of NeuN immunofluorescence were digitized with a ×40 objective within the granular cell layer of the OB (4 images per section and 5 sections per mouse spaced by 336 µm). Within a randomly selected squared zone (constant area) inside the granular cell layer, the total area of immunoreactive profiles was calculated after an image segmentation procedure (binarization) using a constant threshold value of fluorescence signal. The values of immunoreactive areas obtained were expressed as a percentage of the total sampled area and averaged within each experimental group, as performed before [33].

#### Double labeling analysis and quantification

Double-labeled sections were analyzed using a TCS SP2 confocal laser microscope (Leica) at the Centre Commun de Quantimétrie (Université Claude Bernard-Lyon1). The co-localization of markers in individual cells was assessed in 15–30 cells from each animal by performing z-stack acquisitions and three-dimensional reconstructions using the softwares QWin (Leica) and Adobe Photoshop, version CS (Adobe system, San Jose, CA).
